# Caring with time and despite de time: Reflections on prognosis

**DOI:** 10.1017/S1478951525100680

**Published:** 2025-08-22

**Authors:** João Carlos Geber-Junior, Daniel Neves Forte

**Affiliations:** 1 Staff Physician, Center for Oncologic Intercurrence Care (CAIO), Cancer Institute of the State of São Paulo (ICESP), Hospital das Clínicas, Faculty of Medicine, University of São Paulo (FMUSP), São Paulo, Brazil; 2 Postgraduate student in Palliative Care, Faculty of Health Sciences, Sírio-Libanês, São Paulo, Brazil; 3 Intensivist physician and Coordinator of the Postgraduate Program in Palliative Care, Faculty of Health Sciences, Sírio-Libanês, São Paulo, Brazil; 4 Hospital das Clínicas, Faculty of Medicine, University of São Paulo (FMUSP), São Paulo, Brazil

In the corridors of a major oncologic hospital, where precision and technology shape daily decisions, one deceptively simple question continues to echo: “How much time do I have?” No image, biomarker, or score provides a definitive answer. Even the most experienced clinician hesitates – not from lack of knowledge, but from recognition of the profound uncertainty that defines the end of life.

Prognostication in palliative care unfolds between measurable parameters and human presence. Clinical tools offer structure and orientation, but they are insufficient. Some patients who seem close to death live unexpectedly long. Others with favorable indicators decline rapidly. Life, like suffering, resists being fully captured by metrics. As Cassell wrote, suffering is not only physical – it is deeply personal, relational, and moral (Cassel 1982). Prognosis cannot be reduced to probability.

Every estimate of survival is a kind of narrative. To suggest that someone has “days to weeks” is not a neutral transmission of data. It opens space for choices, preparation, and meaning. Prognostic tools, when used without narrative awareness, risk becoming instruments of reduction. They must be used as guides, not verdicts.

Uncertainty is not a failure of medicine. It is the very terrain where the most important work of care occurs. Clinical encounters near the end of life require more than prediction. They demand attunement – to emotional reserves, unspoken bonds, and subtle shifts that no algorithm detects.

Recent qualitative studies describe this experience as a “state of transience” – a reorganization of time, identity, and orientation to the future caused by serious illness (Belar et al. 2024). In these moments, standard language and prognostic boundaries often fail to express what patients and families feel. Clinical presence becomes a way of dwelling within this suspended time.

Some screening approaches embrace this uncertainty directly. The “surprise question” – “Would you be surprised if this patient died within the next year?” – has gained prominence not for its predictive precision but for what it enables: earlier conversations, deeper support, and timely planning. The NECPAL and SPICT tools use this question to catalyze reflection, shifting focus from accuracy to readiness for care.

Quantification, though helpful, can never replace meaning. Tools such as the CriSTAL checklist integrate age, ICU admissions, comorbidities, and other variables to predict short-term death in hospitalized patients (Cardona et al. 2019). These instruments have value – but only when applied with ethical sensitivity and contextual understanding.

The greater risk lies in relying on scores where presence is needed. When assessments substitute for listening, when thresholds replace reflection, the moral core of care is diminished. A recent scoping review confirms that the essence of palliative care lies not in interventions, but in relationships – those moments when clinicians engage with patients as whole persons, not as problems to be solved (Bertaud et al. 2025).

Care that responds to the full complexity of suffering cannot be automated. It requires attention to the unseen: the unspeakable hope, the quiet resignation, the last shared silence. In such moments, the most radical act may be simply to stay.

Even when we cannot predict, we can still be present. And in doing so, we move from measuring time to honoring life. In that gesture, we not only deepen our humanity – we restore the very meaning of healing. As Epstein has noted, when clinicians cultivate mindful presence and shared awareness with patients, they open space for mutual transformation in the face of uncertainty (Epstein 2021).

## References

[ref1] Belar A, Arantzamendi M, Larkin P, et al. (2024) The state of transience, and its influence on the wish to die of advanced disease patients: insights from a qualitative phenomenological study. *BMC Palliative Care* 23(1), 57. doi:10.1186/s12904-024-01380-z 38408953 PMC10895803

[ref2] Bertaud S, Wilkinson D and Kelley M (2025) The heart of palliative care is relational: a scoping review of the ethics of care in palliative medicine. *BMC Palliative Care* 24(1), 150. doi:10.1186/s12904-025-01784-5 40420093 PMC12105298

[ref3] Cardona M, O’Sullivan M, Lewis ET, et al. (2019) Prospective Validation of a Checklist to Predict Short-term Death in Older Patients After Emergency Department Admission in Australia and Ireland. *Academic Emergency Medicine* 26(6), 610–620. doi:10.1111/acem.13664 30428145 PMC6619350

[ref4] Cassel EJ (1982) The Nature of Suffering and the Goals of Medicine. *New England Journal of Medicine* 306(11), 639–645. doi:10.1056/NEJM198203183061104 7057823

[ref5] Epstein RM (2021) Facing epistemic and complex uncertainty in serious illness: The role of mindfulness and shared mind. *Patient Education and Counseling* 104(11), 2635–2642. doi:10.1016/j.pec.2021.07.030 34334265

